# Longitudinal Validation of the Artificial Intelligence Algorithm in Home OCT for Age-Related Macular Degeneration—Report 3

**DOI:** 10.1016/j.xops.2025.100907

**Published:** 2025-08-06

**Authors:** Theodore Leng, Ella H. Leung, S. Krishna Mukkamala, Mehran R. Taban, Moshe Havilio, Kester Nahen, Nishant Mohan, Gidi Benyamini, Tiarnan D.L. Keenan

**Affiliations:** 1 Byers Eye Institute at Stanford, Stanford University School of Medicine, Palo Alto, California; 2 Georgia Retina, Atlanta, Georgia; 3 Macula Retina Vitreous Research Institute, Torrance, California; 4 Notal Vision Inc., Manassas, Virginia; 5 Division of Epidemiology and Clinical Applications, National Eye Institute, National Institutes of Health, Bethesda, Maryland

**Keywords:** Artificial intelligence, Home OCT, Neovascular age-related macular degeneration, OCT, Reference change value

## Abstract

**Purpose:**

Longitudinal validation of the artificial intelligence–based Notal OCT Analyzer (NOA) for identification of clinically significant changes in the trajectories of retinal total hypo-reflective volume (TRO) from daily home OCT (HOCT) self-imaging in eyes with age-related macular degeneration.

**Design:**

Post hoc analysis of the HOCT Fluid Visualization Agreement Study.

**Participants:**

Three hundred seventeen eyes of 180 participants who self-imaged daily using the HOCT for 5 weeks.

**Methods:**

For each eye study, the ground truth of TRO stability or change was defined by human experts grading the 5-week time series of HOCT volume scans. The TRO trajectory of the 5 weeks was plotted separately for each study eye by NOA. Three approaches to identifying the optimal threshold (OT) for a clinically significant change in TRO were pursued: (1) personalized approach based on the reference change value methodology used in laboratory medicine; (2) optimized uniform approach; (3) uniform approach commonly used of 10 volume units (VU). The personalized approach comprised TRO curve-fitting to evaluate the change in amplitude (signal) and within-subject variations (noise), followed by receiver operating characteristic analysis of the signal-to-noise ratio (SNR) to identify the OT for determination of a clinically significant change in TRO.

**Main Outcome Measures:**

Area under the receiver operating characteristic curve (AUROC); sensitivity, specificity, and accuracy at the OT.

**Results:**

Of the 296 trajectories analyzed, 107 (36.1%) were classified as changing and the remaining 189 (63.9%) as stable. The personalized approach had an AUROC of 0.9811 (with 99.1% sensitivity, 89.4% specificity, and 94.2% accuracy), at OT of SNR = 2.42. The optimized uniform approach had an AUROC of 0.9687 (with 94.4% sensitivity, 89.4% specificity, and 91.9% accuracy), at OT of 3.88 VU. The 10 VU uniform approach had 76.6% sensitivity, 94.7% specificity, and 85.7% accuracy.

**Conclusions:**

The NOA-generated trajectories permitted highly accurate determination of TRO change vs. stability, with favorable SNR. The personalized approach had higher sensitivity in detecting TRO change at 99.1% than the uniform approaches. Notal OCT analyzer and its trajectories were a reliable tool for identifying clinically relevant changes in retinal fluid status by the quality standards of laboratory medicine.

**Financial Disclosure(s):**

Proprietary or commercial disclosure may be found in the Footnotes and Disclosures at the end of this article.

Several factors have created an unmet need for home monitoring of neovascular age-related macular degeneration (nAMD) with OCT. Age-related macular degeneration is a prevalent disease with excellent treatment options for the exudative stage.^1^, ^2^, ^3^ These are primarily managed with information collected from in-office OCTs;^4^^,^^5^ however, suboptimal clinical outcomes that fall short of pivotal study results, increasing treatment burden, and high costs suggest that additional advancements are needed.^6^^,^^7^ The Food and Drug Administration recently cleared the breakthrough technology SCANLY home OCT (HOCT) system (Notal Vision, Inc). The system generates near-daily volume scans containing 88 B-scans each. In order to allow the prescribing physicians to utilize such a large volume of information, automated interpretation of the scans was defined as a key element of the system. Volumetric segmentation particularly in the presence of retinal pathologies has been challenging with feature-based analytical image processing methods. In recent years, it has become possible to perform such complex image processing tasks by implementing deep learning-based artificial intelligence (AI) algorithmic methods.^8^^,^^9^ The performance and potential utility of the system have been described previously, and the results of the pivotal studies were recently published in two reports.^10^, ^11^, ^12^, ^13^, ^14^, ^15^, ^16^

Report 1 found that retinal hyporeflective spaces (HRSs), associated with retinal fluid, can be identified on HOCT scans with a high level of agreement with in-office OCT (CIRRUS 5000, Carl Zeiss Meditech).^14^ Report 2 showed that the Notal OCT Analyzer (NOA) AI-based algorithm could segment regions of HRS on the HOCT B-scans, separately for the intraretinal and subretinal compartments, permitting estimation of the HRS volume in a volumetric scan. The study also evaluated the precision of the NOA in calculating the total hypo-reflective volume (TRO) from repeated scans with two HOCT devices during a single session.^15^ The two studies led to the Food and Drug Administration marketing authorization of the first AI-based HOCT system.

The clinical utility of the system for the management of AMD largely depends on its ability to identify changes in the HRSs on OCTs, that is, temporal trends in the TRO trajectories. Broadly, there are only two types of trends—stability or change. Total hypo-reflective volume stability may be seen in eyes whose exudation remains completely resolved with treatment, in eyes with persistent but stable fluid/TRO, or in eyes with non-neovascular AMD. Total hypo-reflective volume change may be seen as either a decrease in response to treatment or an increase due either to new nAMD or to recurrence of exudation as previous treatment wanes.

The problem of detecting clinically significant changes during the monitoring of time-trajectories has been studied intensely in laboratory medicine.^17^ Two methods have been proposed for determining the appropriate reference interval or threshold change value to indicate a clinically significant change. The first is a population-based approach that uses the same threshold interval value for all patients’ trajectories. The main drawback of this method is that there may be individuals who clearly demonstrate a change, although it is numerically below the threshold interval selected for the population. In the context of retinal TRO, an example of a population-based threshold interval is the value of 10 volume units (VU), which has been used as a cutoff to define increased TRO in several studies.^18^

The second, and potentially more sensitive, approach to monitoring individuals uses a methodology derived from laboratory medicine, employing an individualized reference change value (RCV) to define a clinically significant change. The RCV uses an estimate of all the variation in each person’s trajectory to define a personalized interval for each individual. Reference change value is particularly relevant with the advent of personalized medicine. Indeed, personalized medicine is fundamental to the approach of using HOCT to monitor eyes undergoing treatment for nAMD, enabling the physician to tailor treatment plans individually, adjusting to each eye’s response to treatment.^19^^,^^20^

In this study, the RCV methodology was adopted to test the hypothesis that the review and analysis of trajectories validate the longitudinal performance of the NOA in identifying clinically relevant trends in TRO with a high level of accuracy. This would provide additional support to the value of implementing this new technology in clinical research, drug development, and routine retinal care.

## Methods

The HOCT Fluid Visualization Agreement Study was approved by the Advarra Institutional Review Board and conducted in accordance with the Tenets of the Declaration of Helsinki. Written informed consent was obtained from all participants. The clinical trial registration information is publicly available at https://clinicaltrials.gov (NCT04907409). It was conducted at 7 retina clinics in the United States from June 22, 2021, to December 15, 2022.

### The Home OCT System

**Figure 1:**
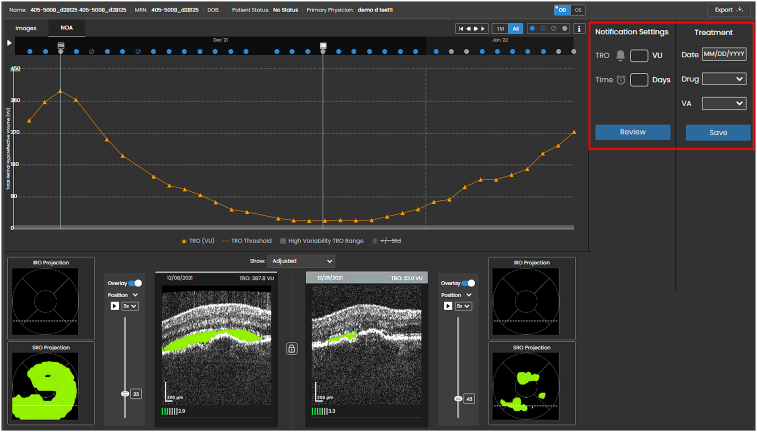
Example of a TRO trajectory for 1 study eye from the 5-week study. The interface where the physician can set the TRO notification threshold is shown in red. TRO = total hypo-reflective volume.

The details of the HOCT system have been previously described.^10^, ^11^, ^12^, ^13^^,^^21^ Briefly, it is a compact spectral domain OCT device that is designed for self-operation at home. It uses a horizontal raster scan pattern of 88 B-scans over a 3 mm × 3 mm retinal area or 10° × 10° field of view. The captured scans are automatically uploaded to the Notal Health Cloud via a built-in cellular modem. Once volumetric scans are reconstructed, they are analyzed by the NOA and are available for the physician to review. Details of the NOA have been described elsewhere, and its validation was described in Report number 2.^13^^,^^15^^,^^22^^,^^23^ The physician interface includes a viewer with two tabs, one for manual scrolling and review of the raw B-scans and the other for reviewing the NOA results, including segmentation, TRO projection maps, TRO volume estimation, and temporal trajectory. Physicians can use a simple interface to set a TRO threshold for notification (Fig 1). The notification mechanism was not used in the two pivotal studies; it is presented here to emphasize the importance of appropriate threshold selection.

### Data Collection

This was a post hoc analysis of the TRO trajectories in the 5-week HOCT Fluid Visualization Agreement Study, which was a prospective longitudinal study. Adults aged ≥55 years with a diagnosis of nAMD in at least one eye and best-corrected visual acuity (BCVA) of 20/320 or better were enrolled. The presence of confounding retinal pathologies was not exclusionary, including epiretinal membranes (ERM), macular holes, pseudocysts, outer retinal tubulations, hemorrhages, pigment epithelial detachments, subretinal hyperreflective material, geographic atrophy, and hyperreflective retinal spots or foci. The participants received a HOCT device delivered to their home, self-installed it, and performed a tutorial and calibration session. Participants were instructed to self-image both eyes at home every day for five consecutive weeks. The output of each daily test was automatically uploaded to the Notal Health Cloud. Volume scans consisting of up to 88 B-scans were reconstructed and were available for review, followed by an analysis by NOA.

The primary goal of the current study was to validate the performance of the NOA in the segmentation and volume estimation of HRS in terms of its longitudinal performance in identifying changes in TRO from the trajectories. In particular, the trajectories allowed the identification of change versus stability in TRO over the 5-week monitoring period, as compared to the change versus stability determined by an expert grader reviewing the time series of all scans. A secondary goal was to compare the performance of a personalized change score to the population-based approach, and specifically to the performance of the commonly used 10 VU threshold, which is equivalent to 10 nL (nL) of retinal fluid.^18^

The RCV approach allows for personalized monitoring of disease progression. It is used to indicate a change that would be unlikely to be due to random variations in the patient measurements, background noise, or artifacts, termed “noise.” One can consider AI-based TRO estimation from near-daily self-imaging with HOCT as analogous to sending out biological samples for laboratory analysis. Similar to laboratory work, TRO estimation includes inherent errors and individual variation. Sources of noise that may affect the quality of images obtained and TRO estimation include the patient’s attention span during the scan, eye positioning and fixation, centration, spacing and uniformity of the coverage of the scanned area by B-scans, diurnal changes or other biological variations, segmentation algorithm inaccuracies, etc. In this context, we adopted that methodology as a broad longitudinal approach of estimating the “noise” level from any possible source over a given trajectory of TRO and comparing it to the “signal” of TRO observed on the same trajectory.

The RCV is defined as RCV = 2^1^^/^^2^ × Z ×SD_T_, where SD_T_ stands for the within-subject random measurement variations from all possible sources, either noise of the measurement device or originated in biological variations, and Z is the number of standard deviations (SDs). Z = 1.96 is typical for laboratory medicine associated with *P* < 0.05.^17^ Hence, a typical reference interval is 2.8 × SD_T_.

For the HOCT trajectories, the SD_T_ and A (amplitude of change or signal) were estimated. The trajectories of TRO were scored by the signal-to-noise ratio (SNR), with the SNR score = A/SD_T_. The SNR score was used to classify the trajectories as either stable or changing. A high classification accuracy would validate the segmentation by NOA since only an accurate estimation of both SD_T_ and A (or their ratio) would allow effective separation between the two classes. A high degree of separation between the two classes for an SNR score threshold of 2.8 would indicate effective identification of change by the standards used in laboratory medicine.

#### Reference Data Set

Two human expert graders, with no access to the NOA segmentation or trajectories, reviewed the volume scans that were collected in 5 weeks of self-imaging of all the study eyes and divided them into two groups—either stable or changing. Cases of disagreement between the two graders were adjudicated by a third grader. Change was defined as a consistent increase or decrease in TRO between any two points in the 5-week study period. This classification was used as the ground truth status of each TRO trajectory.

#### Steps of the Analysis of the Trajectories toward the Personalized Approach:

1. Labeling each of the trajectory data points as belonging to one of three categories of an incline, plateau, or decline period, iteratively resolving questionable points. The labeling function was based on the cumulative-sum generalized likelihood ratio (CUSUM GLR) algorithm.^24^
2. Dividing the trajectory into segments by these three categories.
3. Fit curves represented the visual interpolation process that was performed unconsciously by the reviewing physician while looking for trends. Mimicking that process in the next step, a polynomial fit curve to the trajectory was computed. The fit was calculated for each segment separately, and the continuity was enforced by constraining the nth segment’s fit, to start from segment n-1 fit’s end. The reason for this procedure was that the TRO trajectory was modeled to be continuous, yet each segment may have a distinct pattern, with possibly a discontinuous derivative between the segments, which are typically the result of a reactivation or response to treatment. Figure 2 includes four examples with the original data points color-coded by the trend of their segment, the fit curve, and an indication of the point with the largest amplitude of the change, that is, the signal that follows the above-mentioned constraints.

**Figure 2:**
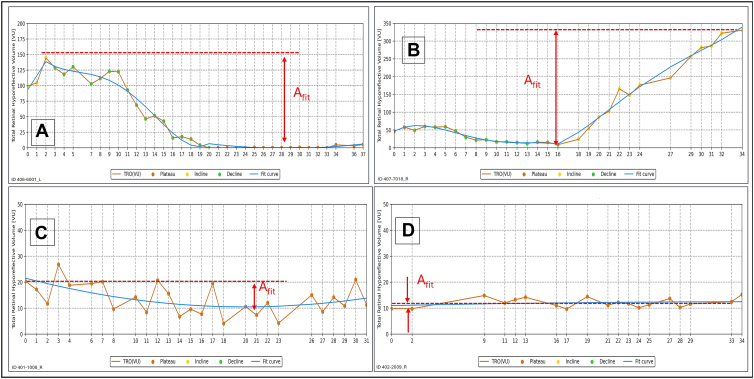
Examples of segmentation and fit (blue curve) to the original trajectory (orange lines) and an indication of the point with the largest amplitude of the change. Example **(A)** shows TRO resolution, example **(B)** shows a reactivation, example **(C)** shows stability with high fluctuations, and example **(D)** shows stability with minimal fluctuations.
4. Calculate the amplitude, that is, the Signal=(Fit curve max – Fit curve min), Noise = $\sqrt{\text{MSE}}$ where $\text{MSE}=\frac{1}{N}\;\sum_{\text{all}\;\text{trajectory}\;\text{points}}{\left(\text{Original}–\text{Fit}\right)}^{2}$ and $SNR\;score=A\;/\;\sqrt{\text{MSE}}$.
5. Sort the trajectories by the SNR score paired with the reference expert grading for each trajectory and plot a receiver operating characteristic (ROC) curve, identify the optimal cutoff value, and calculate the sensitivity, specificity, and accuracy of the SNR score. In addition, the accuracy of the system with a common cutoff of RCV_0_ = 2.8 × SD_T_ was assessed.

For additional context to that approach, Report 2 included the results of the calculation of the repeatability of the HOCT system in the estimation of TRO from eight repeated scans during a cross-sectional study. The relative coefficient of variation was 11.1%, which was superior to the CV of human graders delineating scans of in-office OCT of the same eyes, at 16.4% for eyes with >10 VU of TRO.^15^ The RCV approach used here can be viewed as a longitudinal extension of that method from a special case to the general actual use of the HOCT. The fit curve is an extension of the mean of the repeated measurements while the noise estimate is calculated in the same fashion.

#### Comparing the Performance of a Personalized and a Population-Based Notification Threshold

Based on recent and ongoing research by the Diabetic Retinopathy Clinical Research Retina Network (NCT05904028), HOCT may be set at a population-based notification threshold of increase of 10 VU from no fluid or a persistent fluid level for prompting a physician review.^12^^,^^18^ To cover the range of all possible thresholds, a second ROC was plotted based on the change in amplitude of TRO during the study, A = (Trajectory max – Trajectory min), which was different from the Signal=(Fit curve max – Fit curve min) used in the previous step, as compared to the reference data set. The sensitivity, specificity, and accuracy at the optimal threshold (OT) and at the 10 VU threshold were calculated. This ROC was compared to the curve calculated using the RCV methodology.

The outcome measures were (1) area under the ROC curve (AUROC), OT, sensitivity, specificity, and accuracy of the SNR at the optimal point and at RCV_0_ = 2.8 × SD_T_, for identification of change compared to the ground truth and (2) AUROC, optimal change in VU, sensitivity, specificity, and accuracy for the optimal and the 10 VU population-based thresholds. Statistical analysis was performed using ANACONDA 2024.06/Python 3.12.4.

## Results

A total of 198 participants gave informed consent and were enrolled. Of these, 180 participants (90.9%) initiated testing at home. One hundred and thirty-seven participants tested both eyes and 43 tested one eye, for a total of 317 study eyes. The details of the demographics and baseline characteristics are available in Table 1, Table 2. Briefly, the mean age was 77.1 ± 7.2 years old (range: 55–92 years), 56.7% were women, and most participants were White (96.7%) and non-Hispanic, non-Latinos (98.9%). Based on the designation in Report 1, the mean BCVA was 0.301 logarithm of the minimum angle of resolution (20/40) and 0.234 (20/34) for the primary and secondary eyes, respectively. All primary eyes and 48.1% of secondary eyes were diagnosed with nAMD, whereas 7.3% and 44.5% of the secondary eyes were diagnosed with early and intermediate AMD, respectively.

**Table 1:** Demographics

Baseline Characteristics	Population (n = 180)
Age	
Mean ± SD	77.1 ± 7.2
Median	77.5
Min, max	55, 92
Gender	
Male	78 (43.3%)
Race	
Asian	1 (0.6%)
Black or African American	4 (2.2%)
White	174 (96.7%)
Not reported	1 (0.6%)
Ethnicity	
Not Hispanic or Latino	178 (98.9%)
Not reported	2 (1.1%)
Education	
Less than high school degree	8 (4.4%)
High school degree	43 (23.9%)
Some college (no degree)	47 (26.1%)
College degree (associate or bachelor’s degree)	51 (28.3%)
Graduate degree	25 (13.9%)
Other∗	6 (3.3%)
Study eye	
OD	93 (51.7%)

OD = right eye; SD = standard deviation.

∗ Including some graduate school, trade school, and tech school.

**Table 2:** Baseline Characteristics

Baseline Characteristics	First Eye∗	Second Eye†
	(N = 180)	(N = 137)
AMD diagnosis‡		
Early AMD	0 (0.0%)	10 (7.3%)
Inactive nAMD (no HRS)	92 (51.1%)	35 (25.5%)
Intermediate AMD	0 (0.0%)	61 (44.5%)
Active nAMD (HRS present)	88 (48.9%)	31 (22.6%)
Lens status		
Phakic (cataract present)	65 (36.1%)	47 (34.3%)
Pseudophakia	115 (63.9%)	90 (65.7%)
Ocular media assessment		
Main vessels and the small vessels are clearly seen	179 (99.4%)	136 (99.3%)
Both main and small vessels cannot be seen	1 (0.6%)	1 (0.7%)
Visual distortions		
Present	17 (9.4%)	7 (5.1%)
Absent	163 (90.6%)	130 (94.9%)
Blurry vision		
Present	57 (31.7%)	28 (20.4%)
Absent	123 (68.3%)	109 (79.6%)
Scotoma		
Present	57 (31.7%)	28 (20.4%)
Absent	123 (68.3%)	109 (79.6%)
Prior injections		
Mean ± SD	26.4 ± 26.5	12.1 ± 21.4
Median	17.0	0.0
Min, max	0, 128	0, 125
Spherical equivalent (Diopters)		
Mean ± SD	0.066 ± 1.924	0.044 ± 1.810
Best-corrected visual acuity		
Mean logMAR (Snellen)	0.301 (20/40.0)	0.234 (20/34.3)
SD logMAR	0.251	0.324
20/40 or better	110 (61.1%)	106 (77.4%)
20/41 to 20/80	50 (27.8%)	20 (14.6%)
20/81 to 20/200	14 (7.8%)	9 (6.6%)
20/201 to 20/320	6 (3.3%)	1 (0.7%)
Worse than 20/320	0 (0.0%)	1 (0.7%)
PI assessment of retinal HRS on IO-OCT‡		
Both IRF and SRF	10 (5.6%)	3 (2.2%)
SRF only	40 (22.2%)	13 (9.5%)
IRF only	25 (13.9%)	14 (10.2%)
No IRF nor SRF	105 (58.3%)	107 (78.1%)
Number of eyes included with confounding retinal pathologies§		
Week 1 visit (N = 124)	122 (98.4%)	N/A
Interim visit (N = 47)	47 (100%)	N/A
Week 5 visit (N = 138)	134 (97.1%)	N/A

HRS = hyporeflective space; IO-OCT = in-office OCT; IRF = intraretinal fluid; logMAR = logarithm of the minimum angle of resolution; N = number of eyes with available data; N/A = Not available; nAMD = neovascular age-related macular degeneration; SD = standard deviation; SRF = subretinal fluid.

∗ Primary eye = The study eye in the visualization study.

† Secondary eye = Fellow to the study eyes in the visualization study.

‡ AMD diagnosis based on medical records. OCT HRS status based on investigator review of IO-OCT taken at enrollment visit.

§ Concomitant pathologies Identified by the reading center on in-office OCT scans in the modified visualization analysis populations.

**Figure 3:**
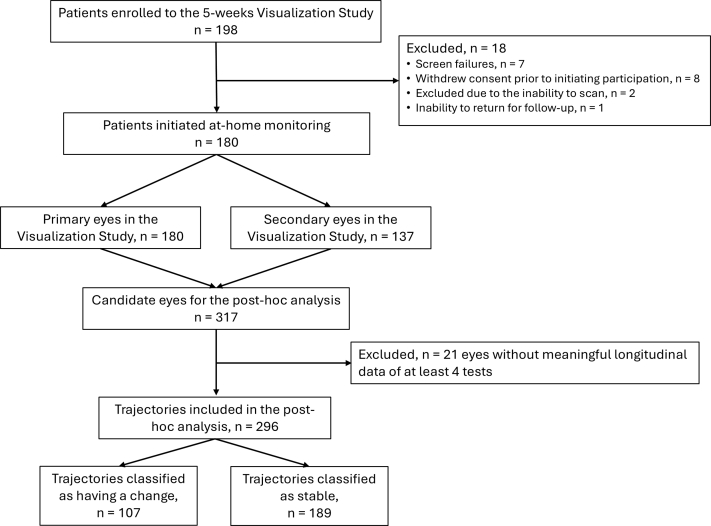
Subjects enrollment and analysis disposition diagram.

After the exclusion of 21 (6.6%) eyes without meaningful longitudinal data (ie, at least 4 tests), a total of 296 eyes’ trajectories were included in the analysis. The participants performed a total of 8242 tests, with a mean (SD) of 27.8 (7.0) tests per eye during a total of 9563 monitoring days, a mean (SD) of 32.3 (6.2) days per eye. Two expert graders agreed on the classification of 280 (94.6%) of the trajectories, and 16 (5.4%) trajectories were classified by a third, adjudicating grader. The final data set included 107 (36.1%) trajectories classified as having a change representing HRS increase or decrease and 189 (63.9%) trajectories classified as stable. These data were used as the reference data set, that is “ground truth.” The disposition of the patients and eyes is detailed in Figure 3.

**Figure 4:**
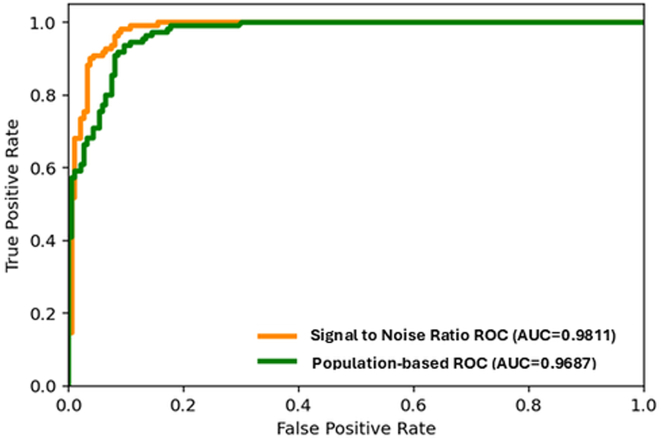
Receiver operating characteristic curves of the signal-to-noise ratio and population-based methods. AUC = area under the curve; ROC = receiver operating characteristic.

**Figure 5:**
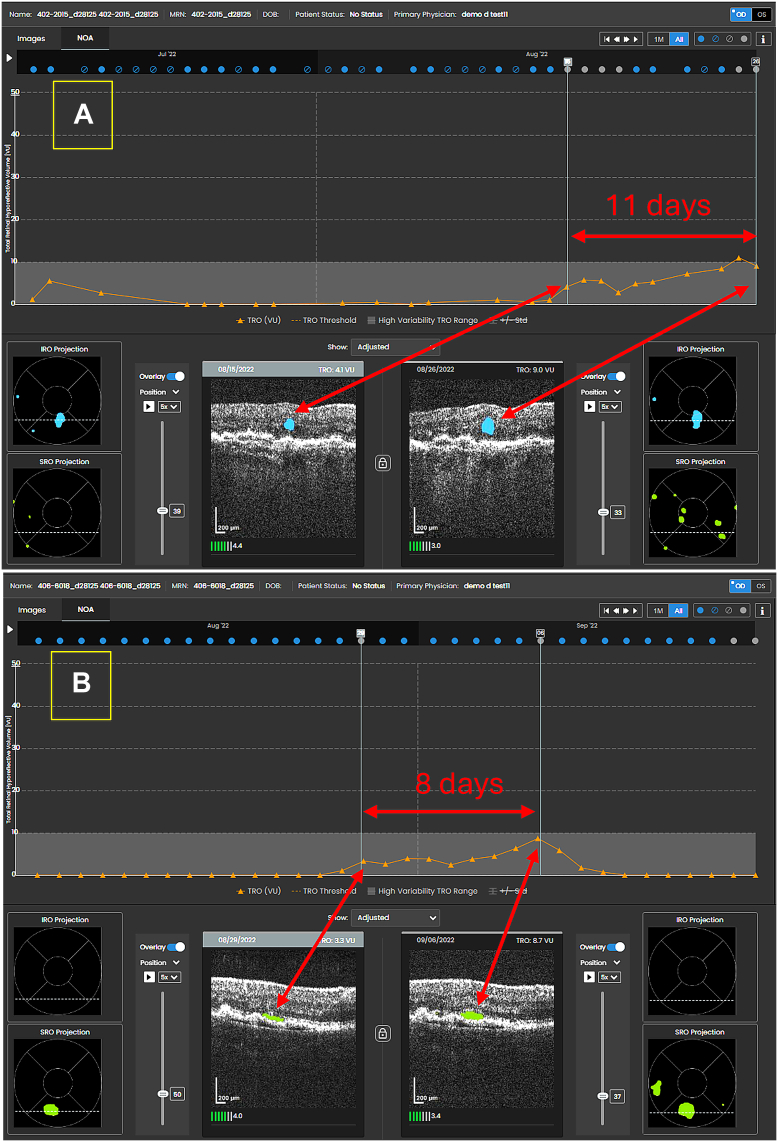
Two examples of reactivations observed by a grader prior to reaching a 10 VU threshold. **(A)** shows an intraretinal hyporeflective space and **(B)** shows a subretinal hyporeflective space. VU = volume units.

The comparison to the reference data set resulted in two ROC curves, presented in Figure 4. The SNR ROC curve had an AUROC of 0.9811 and an optimal SNR score of 2.42, for which the sensitivity was 99.1%, specificity was 89.4%, and accuracy was 94.2%. For reference, similar results were observed with a common RCV of 2.8, for which the sensitivity was 97.2%, specificity was 89.9%, and accuracy was 93.6%. The ROC for population-based thresholds, comparing the maximum to minimum value of the trajectory to the reference data set, had an AUROC of 0.9687, OT of 3.88 VU, sensitivity of 94.4%, specificity of 89.4%, and accuracy of 91.9%. For a threshold change of 10 VU, the sensitivity was 76.6%, specificity was 94.7%, and accuracy was 85.7%. Figure 5 shows two examples of a change identified by a grader before reaching a 10 VU threshold. For case A, HRSs were seen on August 15, 2022, eventually reaching 10 VU on August 26, 2022, 11 days later. For case B, HRSs were seen on August 29, 2022, eventually reaching 8.7 VU 8 days later on September 6, 2022, the time of treatment and subsequent reduction in TRO.

## Discussion

The AI-based HOCT algorithm was able to accurately detect and track the changes in HRS over time in eyes with AMD, as compared to an expert human grader. The fit curve allowed estimation of both the change in amplitude, that is, “signal,” and the trajectory’s random variation level, that is, “noise.” The SNR was able to distinguish between the stable and unstable trajectories, with changes due to nAMD activation or treatment response. The optimal SNR cutoff in the study was 2.42, which was very close to the standard cutoff value of 2.8. Using these thresholds, 99.1% of activations and responses were detectable, with only a small proportion of the stable trajectories being misclassified as a change. These performances were achieved in the presence of confounding pathologies, that by their hyporeflective appearance, have the potential to cause segmentation errors. For example, the area under an ERM may be misclassified as an HRS resulting from the presence of intraretinal fluid. If NOA had not segmented the HRSswith high fidelity, and if the fit curves had not been close to optimal, that is, without major overfit or underfit, the resulting ROC would not have allowed identification of an optimal SNR with acceptable performance. The evaluation of the performance included trajectories at all TRO ranges and was not limited to TROs above a particular threshold. Because the SNR score cutoff was so similar to the acceptable RCV, the NOA-generated trajectories were reliable in identifying clinically relevant changes.

The performance of a population-based threshold demonstrated a lower AUROC. The commonly used threshold of 10 VU had a high specificity of 94.7%, providing for very few false notifications, but a lower sensitivity of 76.6%, missing some reactivations or recovery scenarios that were identified by the graders. An OT for the population-based approach may be approximately 4 VU, but the clinical implications of such a small difference of 6 VU may not be significant, particularly when considering much longer monitoring periods. Setting the notification threshold of the system to be at a more sensitive or a less sensitive level may also have economic implications on the healthcare system driven by the cost of office visits, effect of timing on the long-term treatment efficacy and other parameters.

The RCV approach generated earlier notification than the population-based approach, potentially reducing the time from reactivation to treatment. A practical approach to setting the notification thresholds for eyes that demonstrate robust responses to treatment may be to set the threshold initially at 10 VU, then subsequently adjusting the threshold depending on the individual eye. For example, for case B from Figure 4, while considering the risk of false notifications, the threshold could be adjusted to < 10 VU. The findings also support the potential future implementation of an automated change notification tool that would allow earlier identification of small but significant recurrences in eyes already diagnosed nAMD, or conversions from nonexudative to neovascular AMD, potentially before reaching a population-based threshold of 10 VU. Such a notification tool would require further prospective validation.

The limitations of the study include the post hoc nature of the analyses, the predominantly (96.7%) White study population that is restricting the generalizability of the results across diverse ethnicities and clinical settings and the relatively short study period of 5 weeks. However, that time period was sufficient to observe many episodes of reactivation or resolution of HRS.

In conclusion, the NOA-generated trajectories were accurate in determining stability or change in eyes with AMD. The personalized RCV method was more sensitive than the population-based threshold value of 10 VU in detecting change. Since the RCV threshold identified was comparable to the thresholds commonly used in laboratory medicine, it validated the AI-based algorithms. The ability to monitor for disease recurrence at home could supplement regular in-office evaluations and treatments, allowing for more personalized therapies and potentially decreasing the treatment burden for patients.
